# Hypoxic Status in COPD and ARDS Patients: Impact on Lipid Signature

**DOI:** 10.3390/ijms26136405

**Published:** 2025-07-03

**Authors:** Camillo Morano, Aldijana Sadikovic, Michele Dei Cas, Rocco Francesco Rinaldo, Lorena Duca, Federico Maria Rubino, Michele Mondoni, Davide Chiumello, Sara Ottolenghi, Michele Samaja, Rita Paroni

**Affiliations:** 1 Clinical Biochemistry and Mass Spectrometry Unit, Department of Health Sciences, Università degli Studi di Milano, 20145 Milan, Italy; camillo.morano@unimi.it (C.M.); aldijana.sadikovic@unimi.it (A.S.); michele.deicas@unimi.it (M.D.C.); federico.rubino@unimi.it (F.M.R.); rita.paroni@unimi.it (R.P.); 2 Respiratory Diseases Unit, Department of Medical Sciences, AOU Città della Salute e della Scienza di Torino, Università degli Studi di Torino, 10124 Turin, Italy; roccofrancesco.rinaldo@unito.it; 3 Unit of Medicine and Metabolic Diseases, Fondazione IRCCS Ca’ Granda Ospedale Maggiore Policlinico, 20122 Milan, Italy; lorena.duca@policlinico.mi.it; 4 Respiratory Unit, Department of Health Sciences, ASST Santi Paolo e Carlo, Università degli Studi di Milano, 20145 Milan, Italy; michele.mondoni@unimi.it; 5 Complex Structure of Anaesthesia and Intensive Care, Department of Health Sciences, ASST Santi Paolo e Carlo, Università degli Studi di Milano, 20145 Milan, Italy; davide.chiumello@unimi.it; 6 Department of Medicine and Surgery, University of Milano Bicocca, 20126 Milan, Italy; s.ottolenghi@campus.unimib.it; 7 Department of Emergency, Ospedale San Carlo Borromeo, ASST Santi Paolo e Carlo, 20172 Milan, Italy

**Keywords:** acute hypoxia, chronic hypoxia, plasma lipidome, plasma biomarkers, mass spectrometry, chronic obstructive pulmonary disease, acute respiratory distress syndrome

## Abstract

In patients with respiratory diseases, a panel of markers is often used to assess disease severity and progression. Here we test whether the serum lipid signature may surge as a reliable alternative marker to monitor systemic hypoxia, a frequent unfavourable outcome in acute respiratory distress syndrome (ARDS) and chronic obstructive pulmonary diseases (COPD). We recruited 9 healthy controls, 10 COPD patients, and 10 ARDS patients. Various markers related to inflammation, redox imbalance, and iron handling were measured alongside lipid profiles obtained through untargeted lipidomic analysis. The results show that serum lipids were moderately lower in COPD patients and significantly reduced in ARDS patients compared to the controls. Six lipid classes (cholesteryl esters, coenzyme Q, phosphatidylinositol, sterols, hexosylceramides, and phosphatidylethanolamine) exhibited significant changes (ANOVA *p* < 0.05) and correlated with the Horowitz index (P/F), suggesting their potential as markers of hypoxia severity. While conventional markers also correlated with P/F, the lipid signature was more specific and reliable. This study highlights that hypoxia in pulmonary diseases depresses circulating lipids, with certain lipid classes offering more precise predictions of hypoxia severity. Expanding this research to larger populations could support the lipid signature as a clinical tool.

## 1. Introduction

Impaired gas exchange in pulmonary diseases is often associated with hypoxia, i.e., any situation whereby the oxygen supply to tissues does not meet the demand. In addition to representing a serious challenge for the well-being of patients with respiratory diseases, systemic hypoxia is also an independent factor that often exacerbates the pulmonary dysfunction thereby surging as a critical element in a vicious cycle. Hypoxia may also originate from environmental situations, such as high altitude, but while the body can orchestrate defences against altitude-induced hypoxia through pro-survival responses, pulmonary hypoxia represents a potentially lethal challenge characterized by high mortality and morbidity scores. One example of the dual response to hypoxia is erythropoiesis: while altitude hypoxia increases blood hemoglobin (Hb) potentially enhancing the blood oxygen transport, the pulmonary hypoxia in pulmonary diseases patients is often associated with anemia, likely due to impaired iron handling by hepcidin (Hep) and dysregulated transferrin receptor expression, secondary to inflammation [1].

In this work, we focus on patients affected by acute respiratory distress syndrome (ARDS) and chronic obstructive pulmonary disease (COPD), as examples of pulmonary diseases with a high mortality and incidence of comorbidities. ARDS, an acute condition characterized by the sudden onset of severe hypoxemia without evidence of heart failure or volume overload, has a hospital mortality in the range of 34.9–46.1% [2]. COPD, a chronic lung disease characterized by persistent respiratory symptoms and airflow limitation, due to small airway obstruction and parenchymal destruction [3], is the fourth leading cause of death worldwide, causing 3.5 million deaths in 2021 according to the World Health Organization (https://www.who.int/news-room/fact-sheets/detail/chronic-obstructive-pulmonary-disease-(copd) accessed on 23 March 2025). In both COPD and ARDS patients, hypoxia severity is usually determined by the Horowitz index [4], also known as the PaO_2_/FiO_2_ or P/F ratio, which requires an arterial blood test to be evaluated precisely. Arterial blood sampling is a relatively safe and technically straightforward procedure, but more invasive even for stable COPD ambulatory patients with respect to a peripheral venous sampling [5]. Therefore, the identification of peripheral blood markers that help assess the hypoxic state, disease progression and its outcome, is a key interest in research.

Recently, Dei Cas et al. [6] applied an untargeted approach based on an up-to-date liquid chromatography–tandem high-resolution mass spectrometry (LC-HR-MS) lipid screening technology in severe acute respiratory syndrome Coronavirus 2 (SARS-CoV-2) patients to identify the most frequent lipidome alterations as a function of the severity of the prognostic factors [6]. In that study, patients were clustered according to five clinical signs (inflammation, hypoxia, d-dimer, kidney function, and age), with 7-day mortality unravelling the underlying lipidome signature and identifying three predictive lipid markers. A similar approach applied to healthy subjects exposed to 10-month hypoxia of comparable severity instead revealed a modest decrease in the concentration of all the major lipid classes [7]. However, despite sharing moderate-to-severe hypoxia as a common feature, the pathogenesis found in ARDS and COPD patients is quite different not only from that found in healthy subjects at altitude, but also from that found in SARS-CoV-2 patients [8]. Therefore, focusing on ARDS and COPD requires different discriminating factors and a different rationale.

The aim of the present study is to extend previously obtained omics-based investigations to ARDS and COPD patients with two main objectives: (1) testing whether and how hypoxia impacts the lipidome signature in patients with respiratory diseases, and (2) identifying the potentially compensatory lipidomic responses to a complex situation characterized not only by hypoxia but also by the insurgence of typical disease-associated perturbators such as inflammation, altered iron usage, and perturbed redox imbalance. Comparing ARDS patients to COPD patients would also enable highlighting the differences between acute and chronic hypoxia; while the response to acute hypoxia (ARDS) is expected to be relatively free from compensatory adjustments, the response to chronic hypoxia (COPD) is expected to be at least in part integrated with some compensatory adjustments.

## 2. Results

### 2.1. Subjects and Clinical Assessment

**Figure 1:**
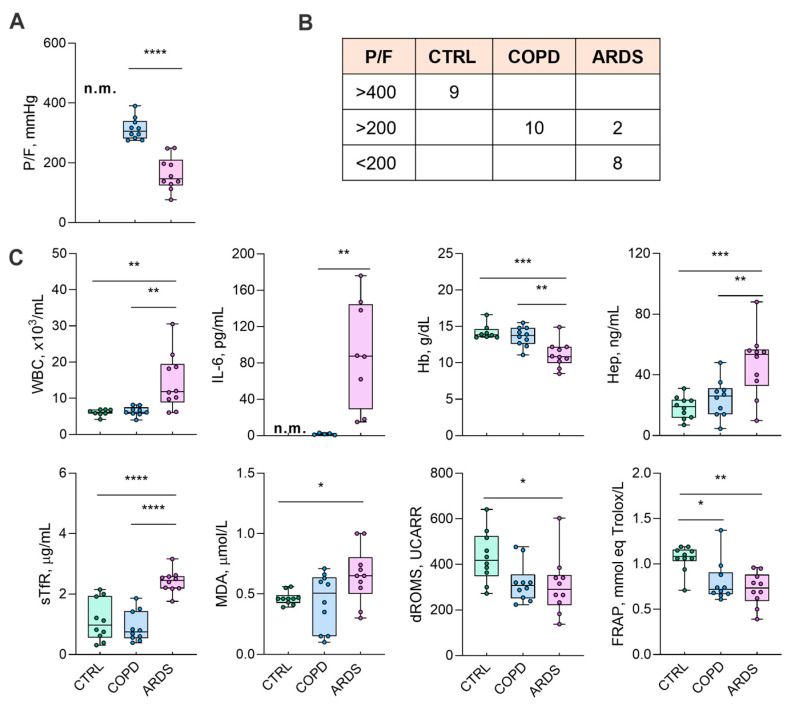
The characteristics of the groups under study. (Panel (**A**)) shows the P/F ratio as a marker of the lung damage due to the underlying disease. (Panel (**B**)) shows the distribution of P/F in the groups, while (Panel (**C**)) shows the biochemical markers. P/F and IL-6 values in the CTRL subjects were not measured (n.m.); a reference value of P/F > 400 was considered for the CTRL subjects according to a peripheral oxygen saturation >95% (Panel (**B**)). Data in (Panel (**C**)) were tested by one-way ANOVA followed by the Bonferroni post hoc test (if ANOVA *p* < 0.05) to check significant differences among groups. P/F and IL-6 data were tested by a two-tailed Student *t*-test (COPD vs. ARDS), excluding any evaluation of the control values. **** *p* < 0.0001, *** *p* < 0.0005, ** *p* < 0.005, and * *p* < 0.05. For abbreviations, see the list of abbreviations.

The 29 subjects investigated in this study were 9 CTRL (5 females + 4 males, average age 65 ± 3 years), 10 COPD (6F + 4M, 78 ± 9 years), and 10 ARDS (6F + 4M, 73 ± 19 years) patients. The trend of the main biochemical markers is reported in Figure 1. We did not measure P/F and interleukin 6 (IL-6) in the CTRL group. However, in healthy CTRL subjects the arterial oxygen saturation was >95%, which suggests P/F > 400 mmHg, and the absence of clinical signs of inflammation suggests IL-6 < 5 pg/mL.

### 2.2. Lipid Alterations

**Figure 2:**
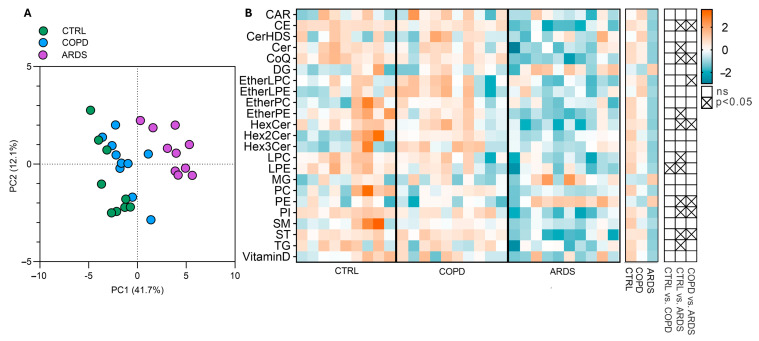
Lipid alterations in the three groups under study. (Panel (**A**)) shows the PLS-DA, that addresses the differences among the groups, highlighting a 41.7% separation on component 1 and 12% on component 2. (Panel (**B**)) shows the intensities of lipid classes as heatmaps for all the subjects (left heatmap) and the group average (right heatmap); signal intensities were Log-transformed and auto-scaled for visualization. The grid on the right shows the significance of the differences between all three groups tested by the ANOVA test followed by the Bonferroni post hoc test.

The PLS-DA profile emerged from the untargeted lipidomic analysis that highlighted a marked separation vs. the lipid signature of the CTRL subjects, especially for the ARDS patients, while the differences between the COPD and CTRL groups were less evident (Figure 2A). The heatmaps in Figure 2B (left and centre) show all the subjects and the group average, respectively, for all the classes of lipids, that were identified and abbreviated as suggested [9]. The emerging trend for most of the lipids corresponds to a progressive decrease in the order CTRL > COPD >> ARDS, but for a few of them (i.e., DG, MG, and PE) the sequence is inverted with ARDS >> COPD > CTRL. Figure 2B (right) shows the direction of the change and whether the change is significant as from the ANOVA analysis followed by the Bonferroni post hoc test. Such analysis reveals statistically significant changes in thirteen lipid classes. Since the primary aim of this study was to identify lipid alterations specific to ARDS, we initially focused on the differences between ARDS patients and healthy controls (CTRL). However, to ensure that the observed changes were not simply due to chronic hypoxia, as seen in COPD, we also compared ARDS with COPD patients. To this end, we selected only those lipid classes that showed statistically significant differences in both comparisons—ARDS vs. CTRL and ARDS vs. COPD—hereinafter referred to as the most significant lipid classes. These include five lipid classes that were decreased in ARDS (CE, CoQ, PI, ST, and HexCer), and one class (PE) that was increased.

As this study aims at discriminating ARDS and COPD patients, we focused on those classes that highlight a statistical difference between ARDS and COPD, hereinafter referred to as the most significant lipid classes. These include five classes of lipids that decrease (CE, CoQ, PI, ST, and HexCer), while PE is increased.

### 2.3. Correlations

**Figure 3:**
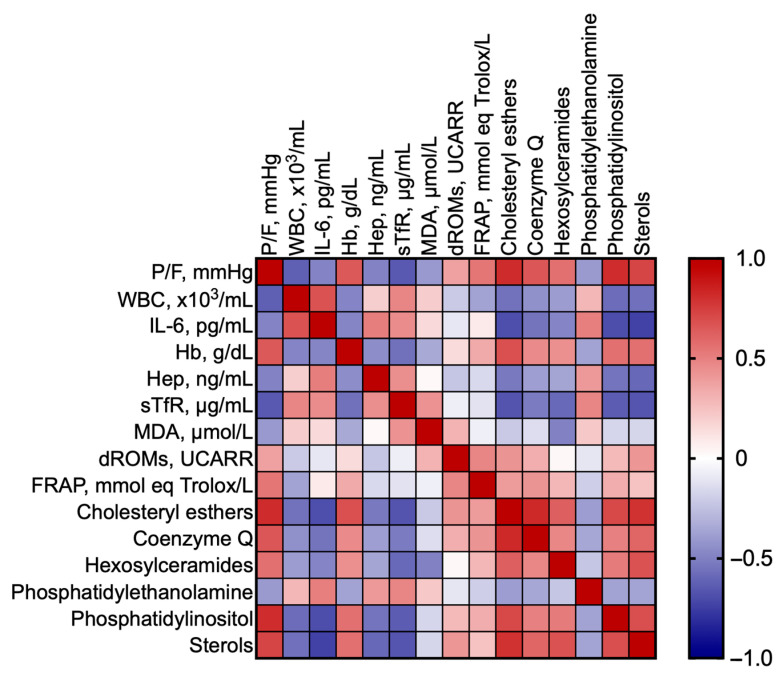
Pearson correlation matrix between each pair of clinical markers and most significant lipid classes.

As a next step, we investigated whether the changes in the most significant lipid classes are correlated with hypoxia severity, best characterized by P/F. Table 1 shows the results of separate correlations between P/F and the biochemical markers, as well as the most significant lipid classes. It appears that P/F is significantly correlated with all the markers, except reactive O_2_ metabolites (dROMs, *p* = 0.0636) and IL-6 (*p* = 0.0867); nonetheless, it correlates with all the most significant lipid classes. Figure 3 shows the Pearson R correlation matrix between each pair of clinical markers and most significant lipid classes.

**Figure 4:**
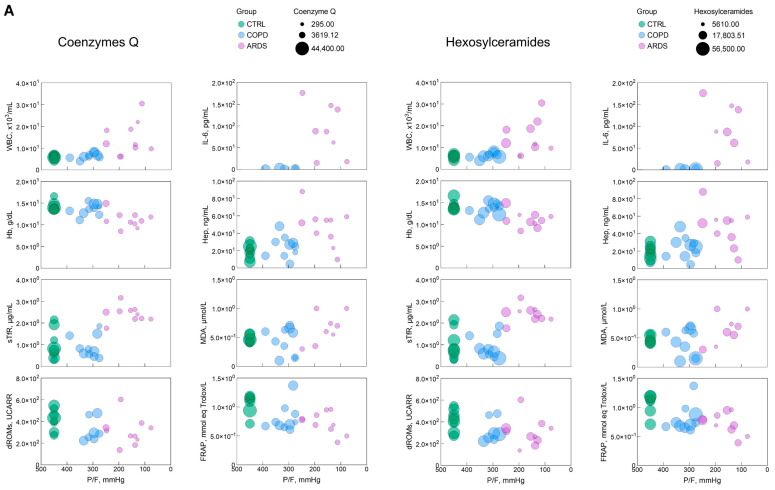

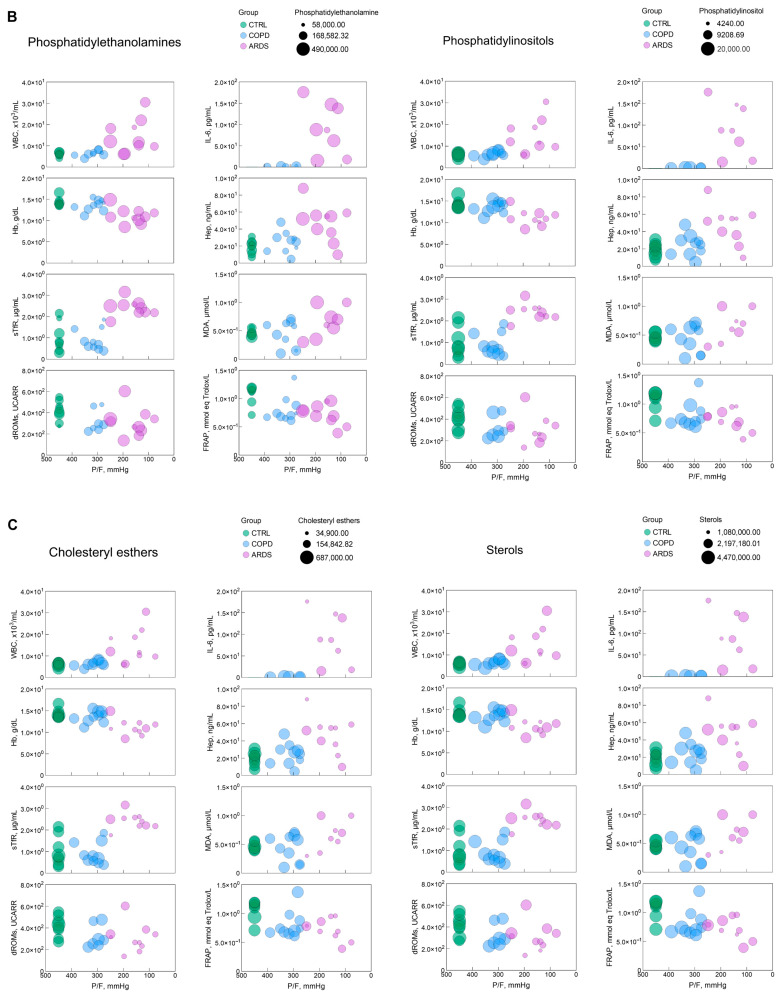
(**A**–**C**) Bubble plots representing the relationship, for each of the most significant lipid classes, between P/F, an index of the hypoxia severity, with each of the clinical and biochemical markers selected in this study. The green, blue, and pink symbols represent CTRL, COPD, and ARDS. IL-6 panels only report COPD and ARDS values, as they were not measured in the CTRL population. The size of the bubble reflects the amount of the relative lipid class as in the key in each panel. The parameters and significance levels of the linear regression fits, which are not reported here for clarity, are shown in Table 1.

To obtain a picture of the interrelationships among the each of the six most significant lipid classes, the various markers of the clinical panel, and P/F, we built bubble plots (Figure 4), i.e., a data visualization method that extends a scatter plot by representing a third variable using the size of the bubbles, thereby enabling the visualization of the relationships among three variables. In these plots, P/F was represented in a reverse way to depict the hypoxia severity along the *X*-axis.

## 3. Discussion

In this study, hypoxia severity, which is assumed to be inversely related to P/F [4], differs markedly in ARDS compared to COPD patients (Figure 1). The difference between COPD and CTRL is less appreciable, in agreement with previous studies where such a difference was either non-significant [10] or moderately significant [1]. We unravel the complex interrelationships among hypoxia severity, the behaviour of eight biochemical markers that here constitute the “clinical panel”, and the lipid signature. Six lipid classes are significantly related to hypoxia severity (Table 1). While five of them (CE, CoQ, PI, HexCer, and ST), are negatively correlated, PE is correlated positively (Table 2).

### 3.1. Clinical Panel

The biochemical markers that constitute the clinical panel were selected based on clinical practice and previous data. White blood cell count (WBC) is commonly used to address the inflammatory state, while IL-6 is an inflammatory marker which is acquiring importance as both a marker and a therapeutic target in ARDS [11]. Blood Hb, Hep, and Soluble Transferrin receptor (sTfR) are used to address anemia and the iron balance [1]. The onset of the redox imbalance is addressed by serum malondialdehyde (MDA) and dROMs, markers of oxidative injury [12], while Ferric Reducing Antioxidant Power (FRAP) is an index of the body’s capacity to counteract injury, and whose depletion reports the exhaustion of the antioxidant power [13]. The clinical panel used in this study differs from that previously used to assess disease progression in SARS-CoV-2 patients [6] because of evident pathogenetic differences. The correlation degree of all the considered markers with hypoxia severity, or the inverse of P/F, is shown in Table 2. It emerges that the statistical significance of the correlation is higher for the most significant lipid classes than for the clinical panel, addressing their better suitability and sensitivity to assess the disease progression.

The bubble plots in Figure 4 help address the 3D relationship between hypoxia severity, the markers of the clinical panel and the most significant lipid classes. In most cases, although not all, such relationships are of the continuous type without depending on the kind of disease. This highlights that the parameters investigated here are essentially unable to discriminate between COPD and ARDS but address the severity of hypoxia independently of the causing disease.

Disrupted lipid metabolism is widely recognized to be linked to hypoxia, inflammation, and the redox imbalance in a vicious cycle. ARDS and, to a lesser extent, COPD, affect the metabolism of lipids, along with carbohydrates and amino acids, amplifying inflammation and oxidative stress [14]. Oxygen free radicals attack lipids on the plasma membrane, further exacerbating lipid peroxidation and free radical formation [15]. While ARDS patients display reduced plasma unsaturated fatty acid levels, especially essential fatty acids, and increased lipid peroxidation [16], the present report focuses on assessing pulmonary diseases based on hypoxia severity. Additionally, data reported here may help understanding if the hypoxia severity-dependent changes may be considered as a part of the hypoxia adaptation process or as an outcome of the deleterious effects of hypoxia.

### 3.2. Lipid Classes

**Sterols** (STs), that include cholesterol and its isomers, and **cholesteryl esters** (CEs), the results of the esterification of cholesterol with long-chain fatty acids, are related to hypoxia in several contexts including pulmonary diseases, where they contribute to the formation of foam cells by macrophages, a feature that is commonly observed in chronic inflammatory lung diseases [17]. Cholesterol biosynthesis is quite expensive for hypoxic cells as it requires eleven oxygen molecules and over one hundred ATP equivalents. Thus, the observed decrease in ST and CE with hypoxia severity may be considered as an obvious response to oxygen lack. The rate of sterol biosynthesis is mainly controlled by squalene monooxygenase, whose expression is controlled by hypoxia that prevents its ubiquitination within a self-defence mechanism that supports ST formation even in hypoxia [18]. Nevertheless, both ST and CE were found to be markedly decreased with hypoxia severity, probably as part of a self-protective mechanisms based on the observation that lower cholesterol levels are accompanied by improved surfactant function [19], due to reduced phospholipid oxidation [20]. This potentially protective chain of events is, however, contrasted by another chain that may be injurious. In fact, increased hypoxia-induced stabilization of the hypoxia-inducible factors (HIFs) may reprogram macrophage metabolism toward increased lipid uptake and the upregulation of ACAT1. This increases the intracellular storage of CEs that are subtracted from the circulating pool but may worsen lung inflammation. Elevated CE levels in bronchoalveolar lavage are indeed being investigated as biomarkers of hypoxic stress in the lungs. In addition, higher CE oxidation mirrors the increase in oxidized LDL and atherosclerotic lesions secondary to inflammation [21]. It is therefore difficult to assess whether the observed decrease in STs and CEs with hypoxia severity is or is not advantageous in hypoxic patients with respiratory diseases.

Although not a member of the most significant lipid classes, **phosphatidylcholine** (PC)-related classes were here observed to decrease proportionally to hypoxia severity (*p* = 0.2, 0.07, 0.01, and 0.007, for EtherLPC, EtherPC, LPC, and PC, respectively). Alterations in PC in the bronchoalveolar lavage fluid of ARDS patients correlate with inflammation and pro-oxidant stress [22], leading to dysfunctional surfactant, lung injury, and inflammation [23]. PC is a substrate for the formation of pro-inflammatory molecules [24]. In agreement with data presented here, previous work addressed lower PC, total-, LDL-, and HDL-cholesterol, but the same TG, in ARDS patients compared to healthy controls, pointing to increased flux through the cytidine diphosphocholine–choline pathway and reduced flux through the **phosphatidylethanolamine** (PE)–N-methyltransferase pathway in ARDS [25]. It remains to be assessed whether the increased PE observed here in ARDS correlates with the lower PE utilization, but the hypoxia-dependent decrease in PC appears advantageous in patients with respiratory diseases under hypoxic stress.

A lipid-soluble antioxidant in mitochondrial membranes and a component in the regeneration of other antioxidants, **coenzymes Q** (CoQs) are related to both hypoxia and redox imbalance. By disrupting the mitochondrial electron transport chain and favouring the release of cytochrome c oxidase, CoQs antagonize hypoxia in preventing oxidative cell injury in several diseases by virtue of its pro-oxidant action [26], but synergizes hypoxia by favouring HIF stabilization [27]. The reduction in CoQ levels observed here is to be related to the depletion of antioxidant potential (FRAP) that is spent to prevent pro-oxidant injury.

Ceramides are known to accumulate in hypoxic cells, which promotes pulmonary endothelial dysfunction and inflammation [28]. The addition of hexoses (glucose or galactose) to ceramides forms **hexosylceramides** (HexCers) that may serve as precursors to the formation of glycosphingolipids, that are less pro-apoptotic than ceramides [29]. In many cells/tissues, hypoxia shifts sphingolipids metabolism toward HexCer synthesis, which is often seen as a cell survival adaptation [30,31]. This shift is favoured by the HIF regulation of glucosylceramide synthase [32]. Thus, the HexCer decrease observed here does not appear to be advantageous to hypoxic patients with respiratory diseases.

**Phosphatidylethanolamine** (PE), a building block of cell membranes, a contributor to the structural integrity of lipid bilayers, and the only lipid class positively correlated with hypoxia severity, plays key roles in the initiation of autophagy [33]. This action synergizes the effects driven by hypoxia, that stimulates autophagy via HIFs in a pathway that requires PE for the conversion of LC3-I to LC3-II, a key step in autophagy [34]. As the recruitment of autophagy is generally felt as a protective feature, the hypoxia-dependent increase in PE appears as an advantageous feature.

**Phosphatidylinositols** (PIs) and their phosphorylated derivatives are well known to be critical mediators of the myriad of pathways that affect cell survival and signalling. Among these, the most prominent is the PI3K/Akt/mTOR pathway [35] that on the one hand triggers the stabilization of HIFs, but on the other hand is inhibited by the redox imbalance. Principally, PIs’ role in inflammation and neovascularization was observed in pathological conditions such as hepatitis B [36]. The observation that, with hypoxia severity, a circulating PI decrease, presumably due to increased tissue utilization, may thus be taken as a phase where the hypoxic drive is superior to the antioxidant power.

### 3.3. Role of Mitochondria

The observation that two of the three major lipid classes of the inner mitochondrial membrane, PC, PE, and cardiolipin [37], are significantly altered in pulmonary diseases strongly supports a pivotal role for mitochondria. Notably, CoQ, a key lipid component of the mitochondrial electron transport chain, also emerged as one of the most affected lipid classes in this study. Mitochondria are central to both acute and chronic oxygen sensing and hypoxic adaptation [38], in part through their regulation of ROS. A detrimental feedback loop is established when damaged mitochondria increase ROS production, which in turn promotes lipid peroxidation and further mitochondrial damage.

The detection of cell-free mitochondrial DNA in plasma has been associated with disease severity and progression in both COPD [39,40] and ARDS [41] patients. Moreover, cell-free mitochondrial DNA levels in plasma may serve as biomarker of anti-inflammatory treatment efficacy in ARDS [42], correlating with pulmonary inflammation and IL-6 production [43]. These findings warrant further investigation into the relationship between cell-free mitochondrial DNA, the lipidomic signature, and the extent of hypoxia.

### 3.4. The Limits and Strengths of the Study

This highlights the need for a developmental phase focused on integrating circulating lipidome analysis into routine clinical and diagnostic practice. Achieving this goal will require concerted efforts by the scientific community to establish standardized workflows and comprehensive guidelines encompassing all stages of the lipidomics pipeline—including preanalytical procedures, lipid extraction methods, mass spectrometric analysis, data processing, and result reporting. Such harmonization is essential to ensure a minimum acceptable standard of data quality and reproducibility, thereby facilitating the transition of lipidomics from research to clinical diagnostics [44,45,46]. Concurrently, it is crucial to evaluate whether lipidomic profiling can effectively stratify patients based on hypoxia severity and whether this stratification yields clinically meaningful benefits. Specifically, further research is needed to determine if such approaches can inform personalized therapeutic strategies, enhance treatment outcomes, or improve prognostic accuracy.

This pilot study is based on a reduced number of subjects of either sex. A power analysis performed before the start of the study suggested that a sample size of n = 8–10/group was suitable for attaining statistically significant data based on the expected changes in the clinical panel. Primarily, because of the low sample size of the tested cohort, we could not perform a statistical analysis based on gender. Further studies with a larger sample size are, however, needed to validate the data reported here in a larger perspective. Arterial PO_2_ has not been measured in CTRL subjects. Therefore, we based the calculation of P/F in these subjects on the peripheral oxygen saturation (>95%).

Using an untargeted LC-MS method, lipids scarcely present in human plasma (e.g., eicosainoids, endocannabinoids, and sphingoid bases) cannot be detected. Nonetheless, the results herein presented might serve as a starting point in future studies based on targeted LC-MS/MS.

The observed changes in the circulating lipidomic profile may likely reflect one or more of the following factors: 1. cellular lipotoxic injury to liver, kidney, and vascular cells; 2. upregulated lipogenesis and downregulated lipid catabolism in key metabolic organs; 3. The enzymatic remodelling of lipoproteins; and 4. organ dysfunction leading to impaired lipid handling and altered plasma lipid signatures. Thus, it remains difficult to assign precisely the observed changes to one or more of these factors. Remarkably, even in pulmonary diseases, plasma alterations represent the outcome not only of the pulmonary dysfunction, but also of the systemic response to the disease condition.

To enable the implementation of the identified panel of significant lipid classes in clinical practice, the data shown in this report should first be confirmed by independent studies and laboratories. After this critical phase, more accessible, cost-effective, and time-efficient analytical methods should be developed in place of LC-MS techniques. Indeed, while highly sensitive and specific, LC-MS methodology is too complex and expensive for routine diagnostic use. This leaves space to a development phase that should focus on design and validation of commercial diagnostic kits tailored for widespread clinical application. These kits would allow for simplified, rapid, and affordable assays, making the analysis of key lipid signatures feasible in most hospital and outpatient laboratory settings. In parallel, it would be important to assess whether the ability to stratify patients based on hypoxia severity using lipidomic profiles will translate into tangible clinical benefits. Specifically, it remains to be determined whether such stratification can guide more personalized therapeutic interventions, improve treatment efficacy, or enhance prognostic accuracy. Addressing these questions will be critical to establishing the clinical utility of lipid-based biomarkers in managing hypoxia-related pulmonary diseases.

One of the strengths of this study is the identification of key lipid classes modulated differently by hypoxia. Thus, the identified lipid signature might help in distinguishing (1) patients with systemic hypoxia due to impaired lung function from (2) healthy individuals exposed to low atmospheric oxygen, such as high-altitude dwellers.

A pilot study performed in subjects dwelling for 11 months at 3600 m in Antarctica has already addressed important issues [7] that need to be validated in a larger population. One of the key factors to be assessed, the occurrence of inflammatory and pro-oxidant factors in COPD and ARDS patients may be potentially confounding in assessing the responses to pathological vs. physiological hypoxia [47].

Another benefit of this study is the perspective reduction in invasive arterial blood sampling to assess hypoxia severity, replaced by venous blood collection. Complications of arterial punctures are rare, but in some cases painful erythematous pulsatile swelling in the wrist, accompanied by a cyst-like collection suggestive of iatrogenic radial pseudoaneurysm have been reported [5].

## 4. Materials and Methods

### 4.1. Patients

This observational study has been approved by the Milan Area 1 Ethics Committee (Prot.N. 5098/2020, 2019/ST/144, revised Prot.N. 0030016, 6 July 2021). We collected clinical data and blood samples from three groups of patients/subjects: (1) ARDS patients, recruited on their first admission to the Intensive Care Unit at the San Paolo Hospital in Milan, Italy, within 24 h after an ARDS diagnosis; (2) stable COPD patients, recruited in the Pneumology Ambulatory at the San Paolo Hospital in Milan, Italy, during routine check-ups if the peripheral O_2_ saturation was <94% and arterial blood gas analysis was included in the visit; and (3) age-matched healthy control subjects, recruited in the laboratory. Exclusion criteria for all groups were age <18 y, and pre-existing chronic kidney disease, SARS-CoV-2 infection, a known history of hematologic or other respiratory diseases. The minimal sample size was established a priori through power analysis for attaining significant (*p* < 0.05, two-tailed) differences based on the expected changes in the clinical parameters cited below, assuming alpha = 0.05 and power = 0.80.

### 4.2. Blood Gas Analysis

Arterial blood samples taken from the radial artery were immediately analyzed in a blood gas analyzer (Siemens RAPIDPoint 405, Munich, Germany). The Horowitz index [4], also known as the PaO_2_/FiO_2_ or P/F ratio, was taken as a marker for evaluating the extent of damage to the lungs, following the indications of the 2012 Berlin ARDS Definition Taskforce [48].

### 4.3. Blood Samples

In total, 12 ml of venous peripheral blood was gathered from the antecubital vein in two vacutainer tubes, with EDTA and with serum separating gel, and centrifuged for 15 min at 3000 rpm within 30 min after collection. Serum and plasma were aliquoted and stored at −20 °C for the biochemical assays.

#### 4.3.1. Hepcidin

The EIA kit for competitive immunoassays from Peninsula Laboratories International (BMA Biomedicals, Augst, Switzerland) was used for the hepcidin-25 assay (Hep). The average reference value is 20 ng/mL [49].

#### 4.3.2. Soluble Transferrin Receptor

The dosage of sTfR was performed in duplicate by enzyme-linked immunosorbent assay (sTfR Human ELISA, Biovendor, Brno, Czech Republic).

#### 4.3.3. Ferric Reducing Antioxidant Power

FRAP analysis enables assessing the body’s capacity to resist an oxidative challenge, e.g., the presence of Fe(III)(2,4,6-tripyridyl-*s*-triazine)2Cl3. A total of 5 μL of plasma was added to 300 μL of freshly prepared FRAP solution [13] in a 96-well plate, incubated at 37 °C, pH 3.6 for 5 min, and the absorbance measured at 593 nm against blank. FRAP was calculated against a standard curve, and expressed in Trolox equivalents, with the reference values of 1.5 mmol Trolox/L [13]. All the reagents were acquired from Sigma Aldrich. The assay was performed in duplicate.

#### 4.3.4. Malondialdehyde

Lipid peroxidation, a marker of the oxidative stress damage, was measured in duplicate by BIOXYTECH LPO-586 Colorimetric Assay For Lipid Peroxidation (Oxys Research, Portland, OR, USA).

#### 4.3.5. dROMs

dROMs, i.e., compounds generated by the interaction of ROS with organic molecules, were measured by Diacron Inc. 25 (Grosseto, Italy), using a control serum with known dROMs concentration as control, in an EnSight™ Multimode Plate Reader (Perkin-Elmer, Monza, Italy), following a 90 min incubation at 37 °C, and reading the absorbance at 546 nm. The d-ROM level is expressed in Carratelli Units (UCARR).

#### 4.3.6. Interleukin 6

IL-6, an inflammatory marker released by activated macrophages and T cells, is known as an acute phase response marker [50] and was assessed by IL-6 ELISA kit (IBL international, Hamburg, Germany).

### 4.4. Lipidome Signature

#### 4.4.1. Chemicals and Reagents

The chemicals acetonitrile, 2-propanol MS-grade, acetonitrile MS-grade, methanol, chloroform, formic acid MS-grade, and ammonium acetate MS-grade were purchased from Sigma-Aldrich (St. Louis, MO, USA). All aqueous solutions were prepared using purified water at a Milli-Q grade (Burlington, MA, USA).

#### 4.4.2. Lipids Extraction

We followed an improvement of established techniques [51]. Briefly, 25 µL of plasma was diluted with water to 100 µL and mixed with 850 µL of a methanol/chloroform (2:1, *v*/*v*), oscillated in a thermomixer (1 h, 800 rpm, 5 °C), centrifuged (25 min, 15,000 rpm), and the organic phase was evaporated under nitrogen. The residues were dissolved in 100 µL of 2:1 isopropanol/acetonitrile+ 0.05% butylated hydroxytoluene (BHT) solution, centrifuged for 10 min at 13,400 RPM, and withdrawn to a glass vial. A total of 5 µL of organic extract was loaded onto the column for each analysis. The addition of BHT during sample preparation prevented unspecific oxidation.

#### 4.4.3. Untargeted Lipidomic

All samples were analyzed at UNITECH OMICs (University of Milano, Italy) using Agilent 1290 Infinity II LC (Agilent, Santa Clara, CA, USA) connected to ZenoTOF 7600 System (SCIEX, Concord, ON, Canada) equipped with Turbo V™ Ion Source with ESI Probe. All samples were analyzed in duplicate in both positive and negative modes with electrospray ionization. Spectra were contemporarily acquired by full-mass scan from 200 to 2000 *m*/*z* and top-20 data-dependent acquisition, with dynamic background subtraction, from 50 to 2000 *m*/*z*. The accumulation times were for TOF-MS 100 ms and for TOF MS/MS 40 ms with a total scan time of less than 1 s. The declustering potential was fixed to ±60 eV, and the collision energy was ± 35 ± 15 eV. Electrospray ionization voltages were set at 5.5 and −4.5 kV. The source and gas parameters were: GS1 55 psi, GS2 65 psi, Curtain gas 35 psi, and a temperature of 500 °C. The chromatographic separation was reached on a reverse-phase Acquity CSH C18 column 1.7 μm, 2.1 × 100 mm (Waters, Franklin, MA, USA) equipped with a precolumn by a gradient between (A) water/acetonitrile (60:40) and (B) 2-propanol/acetonitrile (90:10), both containing 10 mM ammonium acetate and 0.1% of formic acid. The column oven temperature was 55 °C with a constant flow of 0.4 mL/min. Gradient details were the following (%B): 0 min 40%, 2 min 40%, 2.5 min 50%, 12.5 min 55%, 13 min 70%, 19 min 99%, 24 min 99%, and 24.2 min 40%, and were maintained for 30 min for reconditioning.

#### 4.4.4. LC-HR-MS Data Processing

The spectra deconvolution, peak alignment, and sample normalization were attained using the open-source software for untargeted metabolomics and lipidomics data MS-DIAL (ver. 5.4). MS and MS/MS tolerance for peak profile was set to 0.01 and 0.05 Da, respectively. The peak detection was conducted using the following parameters: 250 cps as the min threshold and a 0.1 mass slice width. Identification was achieved by matching spectra with a LipidBlast database or a mass spectral library built in-house with an identification score superior to 70%. The intensities of the analytes were normalized by the Lowess algorithm and those with (1) a CV% superior to 30% in the QC pool sample or (2) a fold-change against the blank sample inferior to 10 were excluded. Mass spectrometry performances during the batch were monitored by the intensities of deuterated internal standard mix (EQUISPLASH™ LIPIDOMIX™ QUANTITATIVE MASS SPEC INTERNAL STANDARD, Avanti Polar, AL, USA).

### 4.5. Statistics and Data Visualization

Multivariate analysis was achieved with MetaboAnalyst 5.0, after data Log-transformation and auto-scaling. Partial least squares discriminant analysis (PLS-DA) was performed to verify and increase the group separation. In PLS-DA, the dimension of a dataset is reduced while retaining as much information as possible; in particular, all the data acquired from a sample are condensed in a single dot, characterized by two dimensions. To further corroborate the data, univariate statistical analysis and other multivariate statistical analyses were performed by GraphPad Prism 10.0 (GraphPad Software, Inc., La Jolla, CA, USA) using one-way ANOVA with a cutoff of *p* < 0.05.

### 4.6. GenAI

In this manuscript, we did not make use of GenAI tools.

## 5. Conclusions

The systemic hypoxia associated with pulmonary diseases leads to a general reduction in circulating lipid levels, with this effect being more pronounced in ARDS patients than in those with COPD—likely reflecting the greater severity of hypoxia. Six lipid classes have emerged as potential indicators of hypoxia severity, showing greater specificity and reliability than conventional biomarkers. Expanding this study to a larger cohort could strengthen the evidence supporting the use of the lipid signature as a clinically applicable diagnostic tool.

## Figures and Tables

**Table 1:** Correlation and linear regression between P/F and each of the biochemical markers belonging to the clinical panel, as well as the most significant lipid classes. The squared correlation coefficient (R^2^) is reported along with the results of the linear regression (slope and Y-intercept with the relative SE) and the significance level of the regression (F and *p* values). For abbreviations, see the list of abbreviations.

		R^2^	Slope	±	SE	Y-Intercept	±	SE	F	*p* Value
Clinical Panel	WBC	0.3937	−0.0309	±	0.00767	18.58	±	2.486	16.24	0.0005
	IL-6	0.2434	−0.3284	±	0.1746	128.7	±	41.46	3.539	0.0867
	Hb	0.4195	0.01027	±	0.00237	9.828	±	0.7643	18.79	0.0002
	Hep	0.2448	−0.07671	±	0.02593	54.07	±	8.498	8.753	0.0064
	sTfR	0.4256	−0.004612	±	0.001051	2.859	±	0.3388	19.27	0.0002
	MDA	0.1635	−0.0007516	±	0.0003546	0.7413	±	0.115	4.494	0.045
	dROMs	0.1417	0.3474	±	0.1783	243	±	57.06	3.798	0.0636
	FRAP	0.2897	0.001046	±	0.0003276	0.5327	±	0.1038	10.2	0.0038
Most significant lipid classes	CE	0.6588	1657	±	229.5	−105,214	±	75,210	52.13	<0.0001
	CoQ	0.433	89.46	±	19.7	−11,472	±	6456	20.62	0.0001
	HexCer	0.3183	58	±	16.33	4795	±	5353	12.61	0.0014
	PE	0.1622	−475.5	±	208	393,418	±	68,179	5.226	0.0303
	PI	0.6483	39.82	±	5.645	2057	±	1850	49.77	<0.0001
	St	0.5151	6705	±	1252	1,029,601	±	410,325	28.68	<0.0001

**Table 2:** A summary of the observed correlations between the hypoxia severity, i.e., the inverse of the Horowitz index (P/F), and each of the parameters considered in this study. The correlations with the less significant lipid classes are not reported here. The ↑ or ↓ signs identify an increase or decrease, respectively. NS, not significant. Four, three, two, and one arrow signs are assigned arbitrarily depending on the *p*-value, *p* < 0.0001, *p* < 0.001, *p* < 0.01, and *p* < 0.05, respectively.

		Correlation with Hypoxia Severity, or Inverse Correlation with the Horowitz Index (P/F)	*p* Value
Clinical Panel	White blood cell count (WBC)	↑↑↑	0.0005
	Interleukin-6 (IL-6)	NS	0.0867
	Blood hemoglobin concentration (Hb)	↓↓↓	0.0002
	Hepcidin (Hep)	↑↑	0.0064
	Soluble isoform of the transferrin receptor (sTfR)	↑↑↑	0.0002
	Malondialdehyde (MDA)	↑	0.045
	Reactive oxygen metabolites (dROMs)	NS	0.0636
	Ferric reducing antioxidant power (FRAP)	↓↓	0.0038
Most significant lipid classes	Cholesteryl esters (CE)	↓↓↓↓	<0.0001
	Coenzyme Q (CoQ)	↓↓↓	0.0001
	Hexosylceramides (HexCer)	↓↓	0.0014
	Phosphatidylethanolamine (PE)	↑	0.0303
	Phosphatidylinositol (PI)	↓↓↓↓	<0.0001
	Sterols (ST)	↓↓↓↓	<0.0001

## Data Availability

The raw data supporting the conclusions of this article will be made available by the authors on request.

## Abbreviations

ARDS, Acute respiratory distress syndrome; BHT, Dibutylhydroxytoluene; CAR, Acylcarnitine; CE, Cholesteryl ester; Cer, Ceramides; CerHDS, Ceramide hydroxy fatty acid-dihydrosphingosine; COPD, Chronic obstructive pulmonary disease; CoQ, Coenzyme Q; DG, Diacylglycerol; dROMS, Reactive oxygen metabolites; EtherDG, Ether-linked diacylglycerol; EtherLPC, Ether-linked lysophosphatidylcholine; EtherLPE, Ether-linked lysophosphatidylethanolamine; EtherPC, Ether-linked phosphatidylcholine; EtherPE, Ether-linked phosphatidylethanolamine; FRAP, Ferric reducing antioxidant power; Hb, Hemoglobin; Hep, Hepcidin; Hex2Cer, Dihexosylceramide; Hex3Cer, Trihexosylceramide; HexCer, Hexosylceramides; P/F, Horowitz index or oxygen partial pressure/inspired oxygen fraction; IL-6, Interleukin 6; LPC, Lysophophatidylcholine; LPE, Lysophosphatidylethanolamine; MDA, Malondialdehyde; MG, Monoacylglycerol; PC, Phosphatidylcholine; PE, Phosphatidylethanolamine; PI, Phosphatidylinositol; PLS-DA, Partial least squares discriminant analysis; SM, Sphingomyelin; ST, Sterols; sTfR, Soluble transferrin receptor; TG, Triacylglycerol; WBC, White blood cells.
